# Discovery of Crystalline Inorganic Solids in the Digital Age

**DOI:** 10.1021/acs.accounts.4c00694

**Published:** 2025-04-17

**Authors:** D. Antypov, A. Vasylenko, C. M. Collins, L. M. Daniels, G. R. Darling, M. S. Dyer, J. B. Claridge, M. J. Rosseinsky

**Affiliations:** Department of Chemistry, University of Liverpool, Liverpool L69 7ZD, U.K.

## Abstract

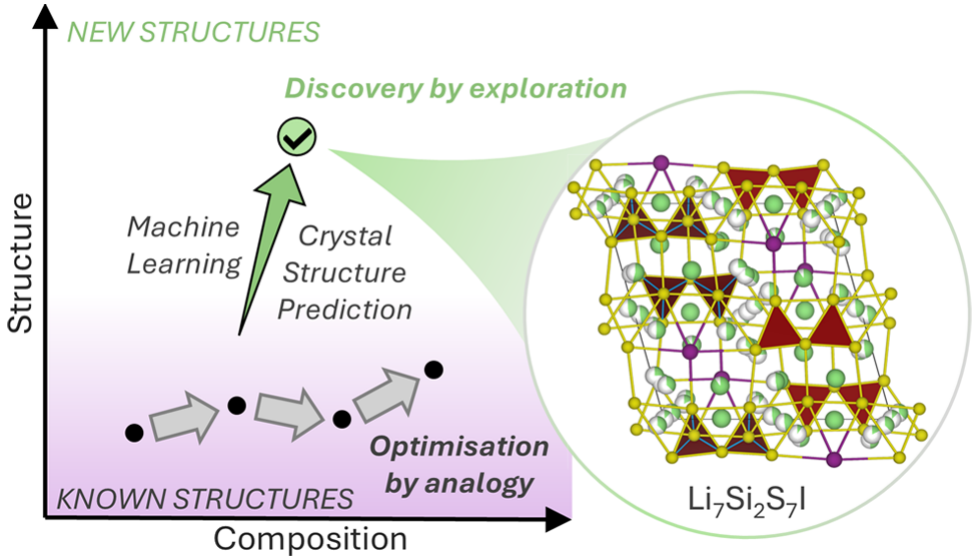

This Account considers how the discovery of crystalline inorganic materials, defined as their experimental realization in the laboratory, can benefit from computation: computational predictions afford candidates for laboratory exploration, not discoveries themselves. The discussion distinguishes between the novelty of a material in terms of its composition and in terms of its structure. The stepwise modification of the composition of a parent material with retention of its crystal structure can reduce the risk in seeking new materials and offers the ability to fine-tune properties which has demonstrated value in optimizing materials performance. However, the parent structures first need to be identified, thus emphasizing the importance of materials discovery beyond simple analogy as a key complementary activity. We describe a workflow we have developed to accelerate discovery of such new structures by addressing many of the challenges, in particular the identification of chemistries that are likely to afford materials and the targeting of reactions within their compositional spaces. Data on experimentally isolated phases are used to prioritise candidate chemistries with machine learning, and crystal structure prediction is used to target compositions within those chemistries for synthesis by computationally constructing probe structures whose energies are indicative of the accessible stability at a given composition. We show how this workflow usefully identifies the parts of chemical space offering new materials and has afforded new structures in practice. The discovery of the solid lithium electrolyte Li_7_Si_2_S_7_I illustrates the role of the workflow in exploring design hypotheses constructed by synthesis researchers and the role of new materials in increasing understanding, in this case by expanding the design paths available for superionic transport. Substitution into Li_7_Si_2_S_7_I affords a structurally related material with superior low temperature transport properties, emphasizing the role of new structures in enabling subsequent materials optimization by compositional modification founded on that structural scaffold.

We contrast our focused hypothesis-driven approach with the recent screening studies that cover a much broader range of chemistries and do not target novel structural motifs. These approaches are good at interpolation and identifying the low hanging fruit for substitutional chemistry, but they struggle to deliver new chemistry knowledge, new understanding and new experimentally observed crystal structures. We comment on reporting the large number of proposed hypothetical structures when considering advances in prediction and the importance of context of the size of the chemical space including continuous composition variation and disorder. An example is the difference between predicting superstructures of known parent structures and experimentally realizing these in the face of competition from structural disorder. Given the scope for prediction of candidates, discussion of structural novelty can usefully be restricted to realized experimental examples based on expert interrogation of their structures. We advocate for bringing experts from chemistry and computer science together to design hypothesis-based routes to materials discovery that incorporate appropriate assessment of novelty.

## Key References

**Figure 1:**
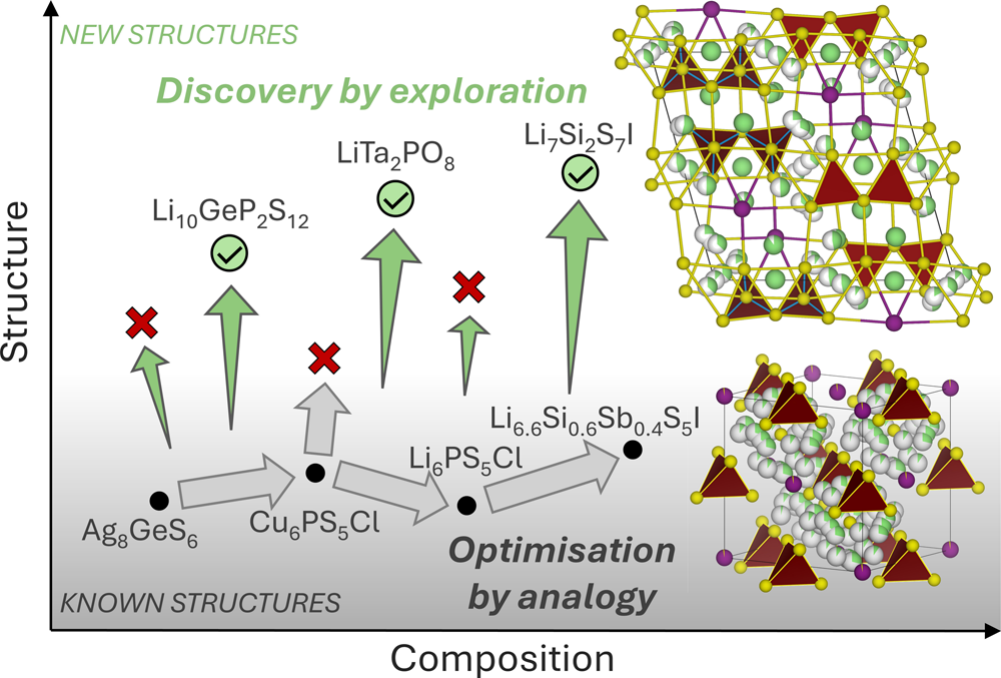
Schematic illustration of two routes to finding new functional materials. Chemical space is represented as the intersection of structure and composition spaces. We use Li-conductors as an example: optimization by analogy with known structures (starting from naturally occurring argyrodite Ag_8_GeS_6_^52^ and making other argyrodite structures with four^33,53^ and eventually five elements^54^ containing Li in their composition) and discovery by exploration that targets new structures (the three materials Li_10_GeP_2_S_12_,^32^ LiTa_2_PO_8_^55^ and Li_7_Si_2_S_7_I^1^ were discovered by exploratory synthesis, not by targeting a specific known structure). The structures shown are those of Li_7_Si_2_S_7_I and the argyrodite Li_6.7_Si_0.7_Sb_0.3_S_5_I. Gray shading represents existing experimental structures.

- HanG.; VasylenkoA.; DanielsL. M.; CollinsC. M.; CortiL.; ChenR.; NiuH.; ManningT. D.; AntypovD.; DyerM. S.; LimJ.; ZanellaM.; SonniM.; BahriM.; JoH.; DangY.; RobertsonC. M.; BlancF.; HardwickL. J.; BrowningN. D.; ClaridgeJ. B.; RosseinskyM. J.Superionic lithium transport via multiple coordination environments defined by two-anion packing. Science2024, 383, 739–74538359130 10.1126/science.adh5115.^1^ The solid electrolyte Li_7_Si_2_S_7_I is discovered by exploring a structural hypothesis connecting anion packings in superionic lithium conductors to intermetallic sphere packings with the workflow described in the text. Its structure illustrates the role of materials discovery in advancing understanding.
- HanG.; DanielsL. M.; VasylenkoA.; MorrisonK. A.; CortiL.; CollinsC. M.; NiuH.; ChenR.; RoberstonC. M.; BlancF.; DyerM. S.; ClaridgeJ. B.; RosseinskyM. J.Enhancement of Low Temperature Superionic Conductivity by Suppression of Li Site Ordering in Li_7_Si_2–*x*_Ge_*x*_S_7_I. Angew. Chem., Int. Ed.2024, 63, e20240937210.1002/anie.20240937238923186.^2^ The modification of the new structure of Li_7_Si_2_S_7_I by substitution of Ge for Si enhances the low temperature lithium ion transport. This represents expansion of composition rather than discovery of a new structure type, as schematically differentiated in Figure 1.
- GusevV. V.; AdamsonD.; DeligkasA.; AntypovD.; CollinsC. M.; KrystaP.; PotapovI.; DarlingG. R.; DyerM. S.; SpirakisP.; RosseinskyM. J.Optimality guarantees for crystal structure prediction. Nature2023, 619, 68–7237407679 10.1038/s41586-023-06071-y.^3^ Unlike heuristic methods for crystal structure prediction that partially explore the potential energy surface of a composition, integer programming is used to guarantee that the exact crystal structure is returned by exploring all possible solutions at once, under clear assumptions.
- VasylenkoA.; GamonJ.; DuffB. B.; GusevV. V.; DanielsL. M.; ZanellaM.; ShinJ. F.; SharpP. M.; MorscherA.; ChenR.; NealeA. R.; HardwickL. J.; ClaridgeJ. B.; BlancF.; GaultoisM. W.; DyerM. S.; RosseinskyM. J.Element selection for crystalline inorganic solid discovery guided by unsupervised machine learning of experimentally explored chemistry. Nat. Commun.2021, 12, 556134548485 10.1038/s41467-021-25343-7PMC8455628.^4^ Machine learning is used to prioritise candidate chemistries for synthesis based on known crystalline materials. Probe structures are calculated within these chemistries to target experimental exploration that lead to the isolation in the laboratory of a new lithium ion conductor.

## Introduction

Materials discovery underpins the development of technology,^5^ and opens new directions for fundamental scientific study across disciplines by providing new phenomena for measurement and analysis.^6,7^ Crystalline inorganic solids are an important subset of functional materials. Structure and composition together control the properties of materials. A common route to improve the functional properties of known materials is to synthesize a closely related analogue differing in composition from the parent while retaining the original structure. Even small changes in composition while maintaining the same structure can have a dramatic impact on properties,^8^ exemplifying the value of this route. There is a much-reduced level of risk in particular regarding the successful synthesis of new compositions when proceeding by stepwise modification of known structures, but these structures did have to be discovered originally – they were either minerals or the product of exploratory synthesis that set out to go beyond existing knowledge, with the associated higher risk. The discovery of new structures is then an important complement to stepwise optimization by analogy, as the distinctive atomic arrangements and associated bonding patterns from such structures offer unexplored routes for the generation and control of properties that may become the next materials family for optimization.

Identifying new materials that are not simple analogues of existing ones is a grand challenge because of the vast size of chemical space. Chemical space is the product of composition space and structure space. Composition space has nearly 100 dimensions and can be continuously variable in solids because of the phenomenon of solid solutions, which are homogeneous phases with a common structure where one element substitutes for another, or vacancies or interstitials are introduced, or a combination thereof.^9,10^ Structural space can have more than three dimensions, and there is no upper limit to the volume of the asymmetric unit (the fraction of the unit cell that allows the rest to be generated by the space group symmetry) or the number of crystallographically distinct sites within it. A single composition can adopt multiple structures (polymorphism), while the same structure types can be formed by different chemistries. Figure 1 schematically represents the complementary activities of the optimization of known structures by analogy, where composition space is enlarged while structure space remains constant, and the discovery of new structures by exploration where both composition and structure spaces are expanded.

We highlight the fundamental difference between essentially unlimited search space for materials structure prediction and the protein folding problem where the primary sequence is given, the secondary structural motifs are well understood and the folding of the tertiary structure can be informed by extensive protein databases. Unlike proteins, inorganic materials lack such hierarchical structural organization and cover much more diverse chemistries. Consequently, no direct analogies or ‘read-across’ should be assumed when addressing the distinctly different challenges of predicting their structures, or the level of impact of one technical approach on both areas.

Materials discovery is the realization of a material in the laboratory. This is subject to both kinetic and thermodynamic constraints.^11^ To synthesize a material, an appropriate synthetic route must be developed—such routes can and often do give access to metastable products, further multiplying the number of possible outcomes.

Given all these challenges, there has been an increasing and welcome focus on the application of computation in materials discovery. Candidate materials may be proposed based on isomorphous (partial or complete) substitution into known structures. If, however, previously unreported structures are the target, then crystal structures need to be predicted and carefully validated and evaluated. Inorganic crystal structure prediction (CSP), the task of computationally identifying the lowest energy crystal structure at a given composition, is now a well-established technique.^12−19^ It has its own practical limitations and constraints, however, given the taxing nature of the problem. For example, the calculations can only treat a finite number of atoms and so cannot accommodate the unlimited cell volume possible for a real crystal.^3,12^

It is important to recognize that the outcome of a computational study, no matter how extensive the range of compositions studied or the power of the approaches and algorithms used, is a set of proposed candidates for experimental evaluation, rather than discovered materials. Failure to appreciate this distinction by the community and society may lead to dramatic consequences for the shape of the research enterprise that slow rather than accelerate materials discovery if appealing high-level narratives that do not address the reality of the challenge lead to an inappropriate balance of technical focus.

The size of chemical space makes the choice of which regions within it to explore for new materials that are not identified by analogy with existing structures particularly consequential. Unless this region is pragmatically and narrowly defined, exhaustive experimental exploration is impossible, considering the range of possible synthetic protocols (which covers a large range of reaction atmosphere, container etc. choices), target compositions, possible starting materials and the combination of such factors. Computational support for the decision to investigate a particular region of chemical space is thus invaluable and needs strengthening.

To do this, it is helpful to consider the choices made by experimentalists when working to discover new materials. Initial selection of the elements to combine delimits the possible synthetic outcomes—this can be formulated as the “how to choose” question and defines a set of elements, referred to here as a phase field, to investigate—a choice of chemistry. Once that choice is made, there is still the question of which compositions to target—“where to look”. With high confidence in selected target compositions, an experimentalist will be prepared to evaluate a range of conditions and develop previously unknown routes to the target, perhaps more so than without such backing. Exploration of reaction and process conditions can benefit from automation and digital optimization.^20−23^

## Materials Discovery Examples

We have developed a workflow that addresses the “how to choose” and “where to look” questions to accelerate the discovery of crystalline inorganic materials. It is not the only possible, or the only sensible, approach but it has been designed to tackle the challenges we have repeatedly encountered in identifying chemistry that can lead to experimentally realized new structures in our own work. By training a machine learning model on the basis of the phase fields that form experimentally isolated compounds in ICSD^24^ (since expanded to a more comprehensive set of databases), we can select unexplored phase fields on the basis of their likelihood of generating experimentally isolatable phases,^4^ using the ability of this variational autoencoder (VAE) to present a ranking of the candidates according to statistical learning from over 200,000 reported examples. As with any machine learning model, these predictions are conditioned by the training data, motivating their expansion by discovery of different chemistry. This information is complementary to expert understanding of structure and bonding because it is based on simultaneous consideration of large amounts of data at a scale beyond that practical for humans.

Within a selected phase field, compositions for study are selected from those with stoichiometries summing to factors of a user-specified maximum number of atoms, initially based on distance in chemical space from known materials,^25^ then by maximizing the information they return about the energy landscape using uncertainty reduction methods such as Bayesian optimization.^26^ At a sampled composition, CSP generates a representative or “probe” structure, most often not the true ground-state structure, because of limits on atom numbers and unit cell sizes imposed to be computationally tractable. The computed energies of probe structures give upper bounds on the ground-state energies for the given compositions, displayed as maps of energetic stability relative to the known stable compounds forming the “convex hull” (the set of compounds comprising the decomposition products of energetically unstable materials).^27^

**Figure 2:**
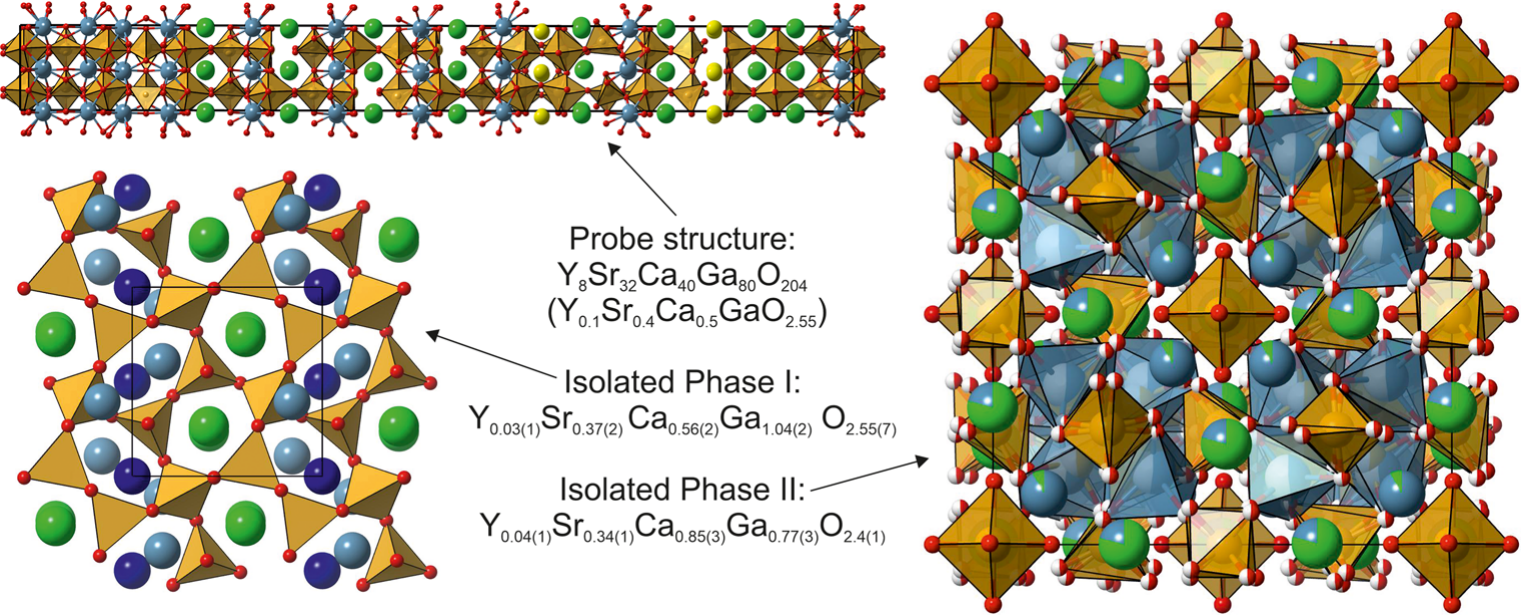
In the probe structure-led exploration of the Y–Sr–Ca–Ga–O phase field,^28^ the identified lowest energy probe structure (top left) led to discovering two experimentally isolated phases. The probe structure (Y_8_Sr_32_Ca_40_Ga_80_O_204_, with composition normalized as Y_0.1_Sr_0.4_Ca_0.5_GaO_2.55_ to contain two cations (Y, Sr, Ca, Ga)) significantly differs from both experimental crystal structures and is compositionally located between them. However, both experimental compounds contain cation-oxygen co-ordination polyhedra found in the probe structure. Atoms colors: Y and Sr (green; diffraction methods cannot distinguish Y^3+^ from Sr^2+^); Ca (light and dark blue); Ga (brown); O (red). In Phase I, two distinct Ca sites are highlighted; in Phase II, site occupancy is shown as pie charts. Adapted with permission from ref (28). Copyright 2017 Springer Nature.

These maps can guide experimentalists to compositions likely to contain thermodynamically stable materials, i.e., the role of the probe structure calculation is to signal promising regions of chemical space for experimental investigation. The Y–Sr–Ca–Ga–O phase field provides an example where this led to successful isolation of two new materials with previously unreported structures (Figure 2), with compositions near to, but different from, those of the probe structures.^28^ Fully characterized, the crystal structures are also different from the probe structures, with large unit cells and extensive site disorder that cannot be captured by reasonable prediction: a dedicated experimental study is needed, where detailed expert assessment of the structural outcomes is essential.

This combination of methods has been successful in discovering compositions^4^ with previously unknown crystal structures,^1,29^ and the targeting can be combined with the predictions of machine learning models for properties of interest to further refine the selection of systems to explore experimentally.^1,4,30^ Given the challenges in experimental exploration, any increase in success rate through such probe structure and properties targeting is welcome. It is however not design because it is not based on a structural hypothesis about the target and of the inevitable limitations of any probe structure in precisely defining structure and composition. It is distinct from discovery, which can be performed by exploratory synthesis in isolation from computation—it is support for discovery, which is intrinsically experimental.

**Figure 3:**
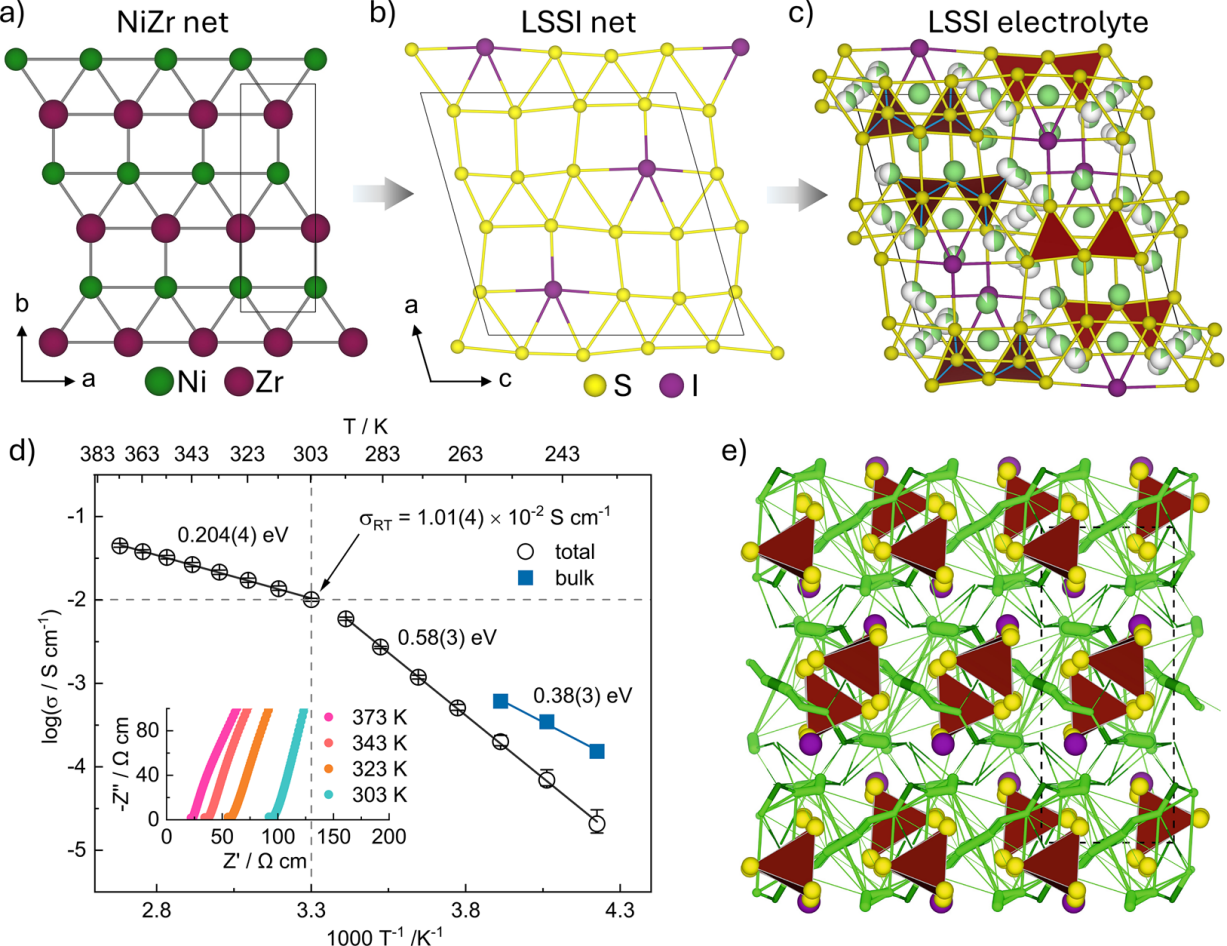
Discovery of the Li electrolyte Li_7_Si_2_S_7_I (LSSI):^1^ (a) 2D net observed in NiZr intermetallic known for hydrogen transport; (b) differently populated but topologically similar net to NiZr produced by the distinct anionic packing in LSSI; (c) LSSI structure accommodates 15 distinct Li environments and supports its superionic transport; the anion net is thus related to NiZr but differently decorated and associated with the 15 lithium sites to afford a new structure; (d) temperature dependence of the total and bulk conductivity of LSSI with derived activation energies shown; (e) AIMD data: calculated Li^+^ pathways are shown as green cylinders whose cross sections are proportional to hopping frequencies. Atom colors: S^2–^ (yellow); I^–^ (purple); Si^4+^ (maroon); Li^+^ (green). Adapted with permission from ref (1). Copyright 2024 AAAS.

Some aspects of design can be introduced through the creation of hypotheses based on chemical understanding. One recent example^1^ is the hypothesis of a connection between the structures of lithium ion conductor anion sublattices and the unconventional (i.e., beyond close packings, bcc and their simple derivatives^31^) sphere packings observed in intermetallics. This is motivated by the occurrence of anion packings in LGPS,^32^ argyrodite^33^ and LLZO^34^ that are connected to such intermetallic structures, and the observation that the interstitial space in such intermetallics can support hydrides that are highly mobile.^35^ This leads to the suggestion that a two (or more) anion net can be used to generate packings akin to those of intermetallics, with the lithium occupying interstitial sites generated by the multiple anion packings as the hydrogens do in the multiple metal packings. The basic chemical requirement for this is Li, two anion formers (*X* and *X*′), and a second electropositive element *M* to stabilize the anion scaffold sufficiently to allow the Li to move through it. VAE ranking of candidate Li–*M*–*X*–*X*′ phase fields affords Li–Si–S–Cl as the highest ranked, but the larger difference in ionic radii between S and I led us to select ninth ranked Li–Si–S–I on the grounds that anion order as in an intermetallic packing was more likely. Probe structure calculations identified a region of composition space containing candidates of sufficiently low energy to motivate synthetic work. This resulted in the discovery of Li_7_Si_2_S_7_I which adopts a unique structure (Figure 3). The five-connected anion network matches that of NiZr, although its decoration by S and I differs from that in the intermetallic. The resulting anion array creates a wide range of interstitial site geometries and supports 15 distinct Li environments. Although current understanding^36,37^ is that a minimal change in coordination geometry over the transport pathway is needed to access high Li conductivity, in fact this wide distribution of geometries affords a conductivity above 10^–2^ S cm^–1^ at room temperature, which is a key threshold for device performance in solid state batteries,^38^ and 3D Li mobility demonstrated by NMR spectroscopy. This arises because the structure creates many inequivalent mobility pathways that all have low energy barriers produced by the anion arrangement, enabling transport between the different sites, as shown in *ab initio* molecular dynamics (AIMD) calculations that quantitatively agree with the measured conductivity. These calculations demonstrate the free energy landscape that the anion array provides for lithium ion motion, identifying 162 unique site-to-site hops with barriers and paths that, while different, are all low due to the role of the varying but effective anion coordination along the transport pathway. A reduction in the number of Li sites occupied on cooling drives a drop in the conductivity and NMR-measured mobility. Though closely related elements form argyrodites, LSSI is a new structure, which demonstrates the potential for discontinuous changes in structure produced by apparently small modifications to composition, reinforcing the inevitability of the probe structure approximation in practice. The structure of LSSI highlights a new direction to obtain high ion mobility in solids, moving beyond disorder between equivalent sites in high symmetry structures to low symmetry cells with many inequivalent sites. These need not act as bottlenecks if the chemistry of the anion scaffold can provide a lower energy barrier pathway, or in fact many such inequivalent pathways, between them to generate a flat energy landscape for lithium motion.

**Figure 4:**
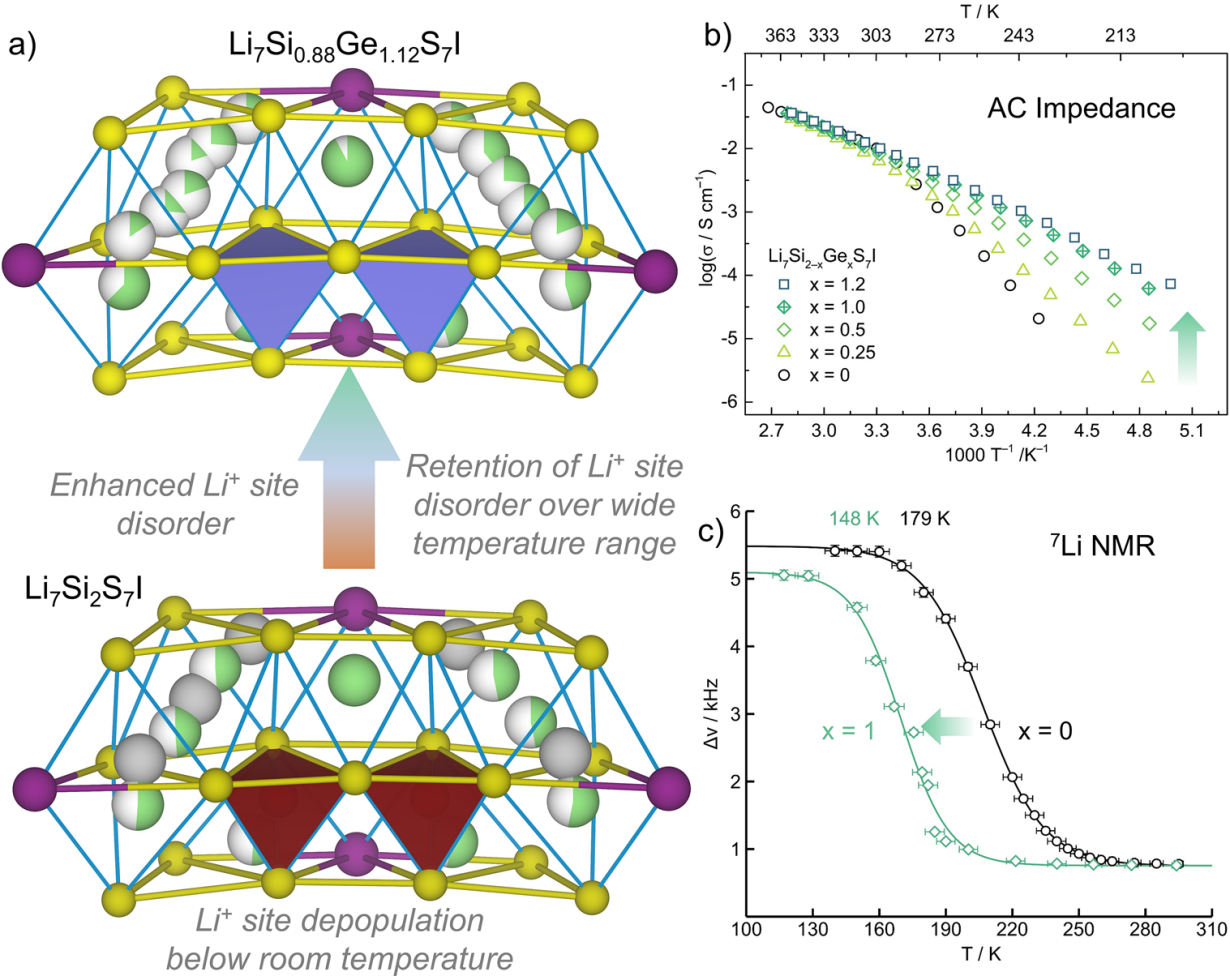
(a) Substitution of Ge for Si in Li_7_Si_2–*x*_Ge_*x*_S_7_I increases Li site disorder compared to LSSI.^2^ This disorder is stabilized to 30 K and enhances the measured Li (b) ion conductivity and (c) mobility (from ^7^Li NMR line narrowing onset temperature) across a wider range of temperatures. Atom colors as in Figure 3, plus Ge^4+^ (lilac). Adapted from ref (2). Available under a CC-BY 4.0 license. Copyright 2024 Wiley-VCH.

Once such a new structure is discovered, this different perspective on structure–property–composition relationships is opened, exemplifying the role of new structures in materials design. Also, the creation of analogues by substitution into the discovered material is enabled—this is a powerful and valuable method to build on the initial discovery, though there is considerable risk in allowing it to become all that we do, or the large majority of it, as noted above. Here substitution of Ge for Si is possible up to 55% and modifies the Li site distribution,^2^ adding a further site and critically stabilizing this large number of sites over the entire *T* range unlike LSSI itself (Figure 4), allowing the high conductivity to persist to low *T* and affording one of the lowest activation energies for long-range Li ion transport measured by ^7^Li NMR spectroscopy in solid-state materials, and thus enhancing the performance of the original material. This is an example of how it is straightforward to expand known structures by analogy through substitution, but the initial identification of such structures, which cannot be by analogy, is an entirely different question (Figure 1). The properties of the analogy-based materials can be superior to those of the initial discovery, highlighting the benefits of discovery, which should not be expected to simultaneously surpass existing figures-of-merit in one bound. The generation of new compositions that are isostructural to known materials can be proposed reliably because of the preservation of structure, which allows tuning and even optimization of properties. This is expansion of composition alone. The synthesis of LSSI corresponds to a new composition and a new structure. The new structural scaffolds arising from such exploratory materials discovery can then be compositionally expanded by analogy to optimize properties, re-emphasizing the importance of their original discovery.

This workflow allows hypotheses to be explored, while recognizing the limitations of both the computational and experimental approaches. Its success can be judged by the realized experimental outcome as it does lead to materials discovery (as with any discovery, it is not possible to claim that it can only be achieved in one way).

## Reinforcing Expertise with Data and Computation in Materials Discovery

It is of course possible to take other paths to benefit from computation. Generative AI is increasingly adopted for proposing crystal structures,^39,40^ which is related to but distinct from identifying the ground state at a given composition. The challenge for generative methods is to form an energetically favorable structure, while extrapolating beyond the crystallographic databases used for training. We recently implemented a method that uses AI for generation of local structure geometries that are then used to initiate physics-driven predictions of materials crystal structure that do target the ground state.^12^ This two-step approach benefits from both the crystallographic average structure data captured by the AI from the training data and from the global optimization that explores the energy landscape with a heuristic using computational chemistry and is not constrained by the structures in the training data.

To complement the range of powerful heuristic models for CSP that only partially explore the energy landscape of a composition, we recently introduced an exact method that,^3^ under clear assumptions, considers all possible structures simultaneously to guarantee to predict the lowest energy outcome. This became possible by formulating the CSP problem for ionic materials as a Quadratic Unconstrained Binary Optimisation (QUBO) problem of atom allocation on a fine grid for which existing powerful solvers can be used. The need for approximations and assumptions persists of course, except now we have guarantees within their limits. As well as reducing the strictness of the current approximations, future developments can use synergies with data-driven and heuristic methods: for example, the interatomic potentials used can be generalized using the data-driven statistical proxy potentials.^41^ The QUBO statement of the problem also allows implementation on quantum computers for CSP that can benefit from this approach to tackling the combinatorial explosion of possible structures.

**Figure 5:**
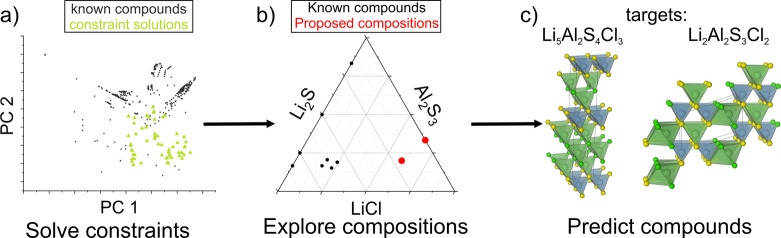
Application of automated reasoning to materials discovery in an automated workflow:^42^ (a) **Solve constraints**: User defines a set of chemical constraints (*e.g.,* minimum EMD^25^ from known compounds, charge neutral, contains a maximum number of atoms). COMGEN then generates compositions compatible with these constraints. (b) **Explore compositions:** For each of the generated compositions, construct the phase field, here LiCl–Li_2_S–Al_2_S_3_. (c) **Predict compounds**: Probe structure prediction calculations with FUSE and prediction of the Li-ion conductivity^43^ for each composition. The energy vs the convex hull (E_hull_ ≤ 45 meV/atom) identifies targets, which are then prioritised according to their predicted Li-ion conductivity. The candidates Li_5_Al_2_S_4_Cl_3_ and Li_2_Al_2_S_3_Cl_2_ are shown. Adapted from ref (42). Available under a CC-BY 4.0 license. Copyright 2025 Wiley-VCH.

To address the “how to choose” question, we have recently introduced automated reasoning^42^ to explore chemical space at scale based on researcher hypotheses: these techniques traceably and exactly build on domain knowledge rather than making inferences from training data. In this work, automated reasoning is applied in the context of constraint solving. We defined a series of chemical constraints (constructed by the domain expert) to specify what we consider a “valid” composition for the exploration in question. Examples of the types of constraints used include charge neutrality, maximum number of atoms in the formula unit when expressed as an integer, selection of starting materials, relative ionic sizes and a minimum distance in composition space from a data set of reference compounds. These types of chemical constraints (for a full list see,^42^ are encoded in the new tool COMGEN, which will identify compositions that satisfy all the constraints set by the user. A user constructs a hypothesis as a series of constraints and explores how varying the constraints affects the resulting compositions, allowing fine-tuning the constraints to address chemistry of interest. We demonstrated the approach by combining COMGEN in a workflow with FUSE^12^ to search for potential Li-ion electrolytes. Our constraint set generated a list of 59 valid compositions (Figure 5a plotted as principal components vs our reference database^43^). For each composition we computed the energy vs the convex hull with FUSE and predicted the Li-ion conductivity using our ML classifier.^43^ The example LiCl–Li_2_S–Al_2_S_3_ phase field is shown in Figure 5b. This resulted in the prediction of eight potential compounds (i.e., candidates, not discoveries), including the two targets shown in Figure 5c. The workflow combines logical reasoning expressed in the solver with pattern recognition in both the ML property prediction and contribution to the CSP before the heuristic potential energy landscape exploration.

## Evaluating Novelty in Materials Discovery

The above examples represent how we encode expert knowledge into our computational workflow – both from chemists in formulating the problem, defining the all-important experimental work and evaluating the outcomes, and from computer scientists in assisting its solution with the appropriate digital tools built collaboratively by the team. We emphasize that expert evaluation is needed to assess the novelty and impact of the discovery, as exemplified by LSSI. To give another example, probe structure calculations, coupled with composition-based machine learnt property models, led to the discovery of Ba_10_Y_6_Ti_4_O_27_ by targeting the correct region of composition space for synthetic exploration.^30^ Although this composition space had been extensively and expertly investigated previously, the computational guidance provided the support needed for the decision to investigate a broader range of synthetic conditions and thus isolate this metastable phase. Ba_10_Y_6_Ti_4_O_27_ is the first bulk oxide quasicrystal, and its unique high-dimensional structure generated record low thermal conductivity for an oxide. The complexity of this material highlights that it can often be impossible to predict the exact composition and/or structure computationally in general.

Although the computational tools above can involve assessing large numbers of candidate structures and compositions, use of raw numbers of such candidates to make claims regarding the nature of any associated advance should be treated with caution. As a periodic calculation requires exactly one site for each atom in the unit cell composition, substitutions of one atom for another on the same crystallographic site leading to disorder and solid solution in the experimental material must necessarily be represented by a larger or lower symmetry unit cell computationally in order to generate separate sites for the two atoms involved – the appropriate size or symmetry of such a cell is unknowable *a priori* and thus the source of approximation, quite reasonably but also unavoidably. This is not a question of terminology, or of disorder being a challenge for theory and experiment (true though that is), rather it reflects that the approximations made prevent a direct connection to discovery.

When evaluating transition metal substitution into the cation sites of the anion vacancy ordered perovskite YBa_2_Fe_3_O_8_, we built computational models that inevitably lowered the symmetry to assess the energetic feasibility of substitution.^44,45^ These models identified which of the range of transition metals considered could be substituted experimentally for Fe into the parent compound. Candidate substituted structures were constructed, which required artificial symmetry lowering and cell expansion together with approximation of the substitution patterns considered, and their energies computed. A simple statistical mechanics model led to the prediction that the substitutions were indeed feasible but that at the required synthesis temperatures they would afford a disordered outcome with no single substitution pattern predominating. The substituted material was disordered by site mixing in its crystallographically determined average structure and did not display the superstructures generated within and required by the computational models used. No new structure thus resulted, reflecting the need to consider multiple disordered outcomes when investigating substitutions into a known structure.

Superstructure generation from currently known scaffolds is a subset of the challenge of materials discovery. The ability to calculate large numbers of these decorated superstructures is impressive and useful in assessing competing local orderings in a disordered material, given assumptions about relevant cell size. Since ICSD explicitly reports the presence of structural disorder and is based on experimentally realized materials, it is not comparable with lists of candidate materials based on different assumptions: such candidates could offer insight into substitution into known scaffolds. Any arising structures should then be seen in the appropriate context given the scale of potential compositions of crystalline solids, and the role of disorder, as highlighted by Cheetham and Seshadri.^46^ With a region of solid solution A_1–*x*_B_*x*_, even a very narrow compositional range can correspond to an unquantifiable number of possible compositions (depending purely on the granularity imposed on composition space), and so even numbers that may appear large to humans do not equate to significant advances in knowledge of chemical space. Given these approximations, studies that predict large numbers of materials computationally using data-driven approaches can be seen as providing probe structures that can be used in understanding the connection between composition and stability, rather than material discoveries. An example of the large numbers that can arise from isovalent substitution into ordered crystal structure prototypes known in ICSD is the proposed 32,598,079 initial candidates that led to the synthesis of Na_*x*_Li_3–x_YCl_6_,^45^ reported as a solid solution between the known end members; Li_3_YCl_6_ (*P*3̅*m*1) and Na_3_YCl_6_ (*R*3̅).^47,48^ Similarly, evaluation of 30,000 AI-generated candidates led to experimental realization of a disordered variant of the rutile structure.^40^ In the SI, we give examples of the distinction between generating large numbers of structures and compositions and the amount of knowledge that this affords beyond what is already known. In any computational search for new materials, instances that lie beyond the approximations made computationally are just as possible experimentally as those that lie within the approximations, and there may be far more of them. The definition of progress should then revert to experimentally realized and clearly understood examples where the connection to other materials and structures is evaluated by experts and explicitly expressed.

**Figure 6:**
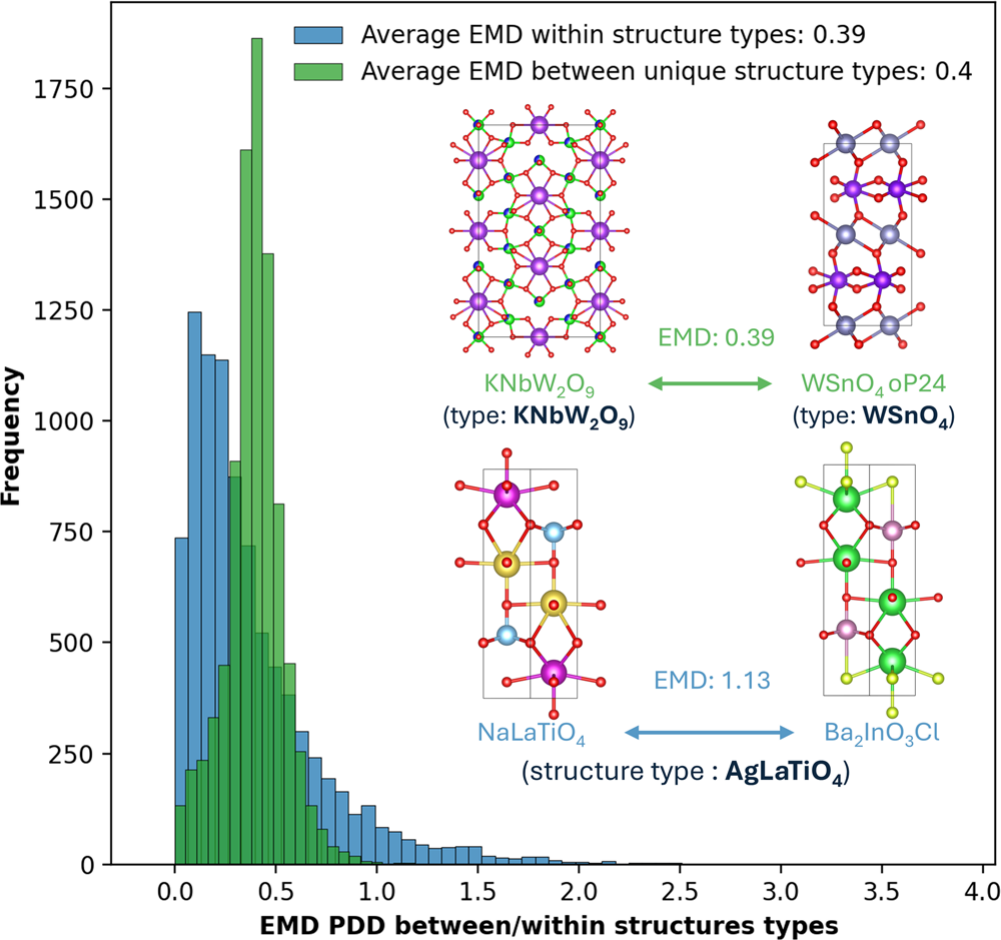
Average Earth Movers’ Distances (EMD) between 8984 unique structure types in ICSD represented as Point-wise Distance Distributions (PDD) (green chart) are comparable to the average EMDs between structures with the same structure type (blue chart).

Given the challenge of modeling disorder, it is important to note the difficulty in assessing novelty of structure types (or prototypes) for structures produced by enforced symmetry reduction from known structures. For example, 45,500 novel prototypes were claimed using threshold-based XtalFinder to count distinct Wyckoff sequence and symmetry group combinations previously unseen in known structures.^49^ As demonstrated by YBa_2_Fe_3_O_8_ above and by ReY_2_B_6_-type structures discussed in the SI, this prototype counting can lead to a combinatorial explosion when applied to artificially ordered structural models (Figure S1), and risks generating many experimentally inaccessible prototypes that likely correspond to solid solutions. Additionally, prototyping lacks a continuous metric to relate structures, creating scenarios where closely related structures are defined as distinct despite clear similarities. There are threshold-free metrics, such as the Earth Mover’s Distance (EMD) on Pointwise Distance Distributions (PDD),^50^ that are provably continuous and powerful to identify exactly equivalent and near-duplicate structures.^51^Figure 6 shows that the spread of this metric within materials adopting the same ICSD structure type is larger than that between materials that adopt different structure types, highlighting the difficulty of automated computational delineation between structure types.

Given these challenges, novelty claims should not rely solely on single metrics and instead should be argued based on multiple factors, standing up to expert scrutiny in logical multistep comparisons. Flowchart-based methodologies, as implemented in StructureMatcher with Framework comparator can be useful in addressing this issue. However, when evaluating novelty, it is crucial to remain aware that having a new composition that has the same structure as another material is not equivalent to finding a new structure, ensuring that synthetic validation and expert assessment remain the ultimate benchmarks for materials discovery. This is exemplified by the CrO_2_–TaO_2_ solid solution case in ref.^40^ which adopts the well-known rutile structure, and passed the StructureMatcher composition novelty test, but not its Framework novelty test. LSSI is a novel framework not simply because it is classified as novel by both of these metrics (and PDD EMD), but because of the expert assessment of its chemistry expressed in the structure itself and the new opportunities it opens for materials design.

## Summary and Outlook

Given the size and complexity of the chemical space of crystalline inorganic solids, it is important to place reported advances, including raw numbers, in context, and understand the level of certainty that can be attributed to them. Scale alone is not a guarantee of value. Any new computationally produced ordered candidate structure and the associated composition can only ever be a proposal, rather than a discovery. Claims of progress purely based on numbers of instances of such structures (or their hypothetical structure types) are hard to assess. It is rather the associated growth in understanding that is important. Any additional predicted stability and structure information is valuable when considered as a probe structure but is distinct from the discovery of a material. When a material is discovered, there is a reason to invest the time required to appropriately place its structure in context by evaluating in detail its relationships to other structures and computing its properties where suitable physical models exist, as we did in the LSSI case. Conversely, there are many predicted structures of low energy that do not turn out to be realizable. Expert assessment of new structures realized experimentally is thus indispensable in claiming advances in understanding – the development of improved metrics to support this process will be valuable.

Materials discovery requires experimental realization. Computational tools offer predicted candidates and thus practical support for key decisions in experimental workflows, both those targeting derivatives of known structures and those seeking new structures, with no single tool representing a definitive solution to the challenge of discovery. However, given the provisional nature of these computed low-energy structures, it is reasonable to focus resources on the synthetic exploration of corresponding regions of the phase fields where stable materials are likely to be found rather than assume that such materials might exist. The questions raised in this Account are not trivial matters of terminology, rather they indicate the appropriate place for and role of different types of computational study in the materials discovery process.

## Supporting Information Available

Examples of enforced symmetry reduction in computed GNoME structures and realized structural novelty (PDF)
