# Supplementary material for: Discovery of Crystalline Inorganic Solids in the Digital Age
Source: Acc Chem Res. 2025 Apr 17;58(9):1355–65. doi: 10.1021/acs.accounts.4c00694 (PMC12060266; doi:10.1021/acs.accounts.4c00694)
Supplement: Supplementary file 1 — ar4c00694_si_001.pdf [file ar4c00694_si_001.pdf]

Supplementary information

Discovery of Crystalline Inorganic Solids in the Digital Age

D. Antypov, A. Vasylenko, C.M. Collins, L.M. Daniels, G.R. Darling, M.S. Dyer, J.B. Claridge, M.J. Rosseinsky*

Department of Chemistry University of Liverpool L69 7ZD UK

* corresponding author email: M.J.Rosseinsky@liverpool.ac.uk

Examples of enforced symmetry reduction in computed GNoME structures and realised structural novelty.

Evaluation of the reported 377,156 unique compositions in the GNoME database shows that there is a preponderance of compounds containing two or more of the lanthanide elements (121,175 structures have two or more lanthanides or Y i.e. 31.4% in GNoME vs 0.12% in ICSD). Lanthanides have very similar chemistry and are often substituted for each other on the same crystallographic site in the parent single lanthanide structure and thus will often form the same structure experimentally, even if multiple lanthanides are present. Thus although there are 1,568 variants of Sn_4La_5 reported as new compositions due purely to multiplication of lanthanide occupancies, this does not reflect the information added, which to the materials chemist is limited. Simple aggregation of multiple lanthanide compositions in this way and removal of multiplied compositions gives 182,553 unique compositions, a 51.6% reduction from 377,156 unique compositions before Yttrium and lanthanides were grouped together.

Moving from composition to structure, there are associated claims of large numbers of new structural prototypes, which again require assessment in context. For example, ICSD structure type ReY_2B_6 adopts orthorhombic space group *Pbam,* with four formula units and 36 atoms in the unit cell giving cell composition of $Re_4Y_8B_{24}$. The GNoME dataset contains 6,658 structures based on elemental substitutions at the sites defining this structure, corresponding to four space groups with the same 36 atoms per cell but expanding beyond the original *Pbam* to two type 1 subgroups ($P2/m$, $Pmc2_1$) and *Pm*, which is a subgroup of $P2/m$ (Figure S1). These symmetry reductions are readily identifiable either from International Tables for Crystallography[1] or the Bilbao server,[2] and correspond to 95 different combinations of space group and occupied symmetry-distinct sites in the unit cell (referred to as Wyckoff positions). None of these are experimentally demonstrated orderings, which illustrates the difference between proposing a new ordering and discovering a new structure. Experimental work on the ReY_2B_6 structure type suggests the ready formation of solid solutions with disordered substitution of multiple elements on the sites within the original *Pbam* structure, such as $Sc_{2.28}Mn_{0.72}B_6$,[3] $Lu_{1.34}V_{1.66}B_6$[4] and $Er(V_{0.77}Ta_{0.23})VB_6$,[5] illustrating the competition with disordered phases that the proposed new ordered structures will face in practice: any proposed ordering of this type faces the strong risk of being disordered when actually made, emphasising that a prediction is a

candidate not a discovery. These 95 proposed new structure prototypes are all candidate orderings, but as the competition with disorder cannot be assessed (for example because approximants with larger cells are not considered, let alone other new prototypes and their disordered derivatives, unaccounted-for disordered derivatives of known compounds, etc.) they cannot be claimed as new structures. If they were synthesisable but disordered in *Pbam*, they would be new compositions isostructural to examples already in ICSD.

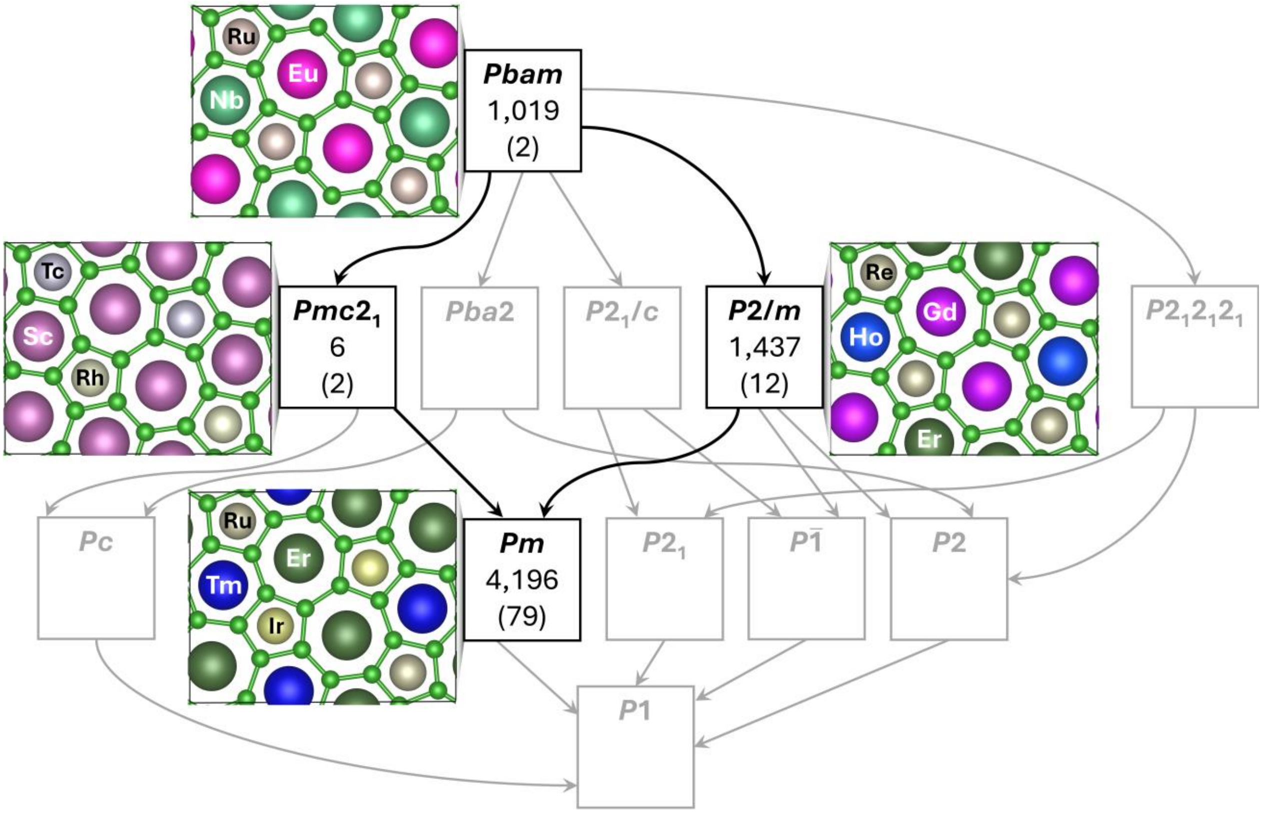

Figure S1. 6,658 derivatives of the ReY_2B_6 structure type reported in GNoME, classified using the Bilbao Crystallographic Server.[2] The subgroups accessible by reduction of symmetry from the parent *Pbam* structure. As the symmetry of the parent cell is reduced from *Pbam* to *Pm* (arrows) retaining the same number of atoms in the unit cell, different decorations of Re (smaller spheres) and Y (larger spheres) sites are explored (B connected green spheres). The number of derivatives within each space group in GNoME is shown, with the number of distinct Wyckoff strings in brackets.

References:

[1] Aroyo, M. I.: *International Tables for Crystallography, Vol A., Space-group symmetry 6th ed.*; John Wiley & Sons: New Jersey, 2016.

[2] Ivantchev, S.; Kroumova, E.; Madariaga, G.; Perez-Mato, J. M.; Aroyo, M. I. SUBGROUPGRAPH: a computer program for analysis of group-subgroup relations between space groups. *J. Appl. Cryst.* **2000**, *33*, 1190-1191.

[3] Mikhalenko, S. I.; Babizhets'kii, V. S.; Kuz'ma, Y. B. The Sc-Mn-B system. *Powder Metall. Met. Ceram.* **2005**, *44*, 567-572.

[4] Kuz'ma, Y. B. Phase Equilibria in the Lu — V — B System and the Structure of the New Boride $Lu_{1.34}V_{1.66}B_6$. *Powder Metall. Met. Ceram.* **2002**, *41*, 162-168.

[5] Kuz'ma, Y. B.; Prots, Y.; Grin, Y. Crystal structure of erbium vanadium tantal boride, $Er(V_{0.77}Ta_{0.23})VB_6$. *Z. Kristallogr.* **2003**, *218*, 159-160.
